# Prevalence of sleep quality disorder among Iranian drivers: a systematic review and meta-analysis

**DOI:** 10.5249/jivr.v10i1.993

**Published:** 2018-01

**Authors:** Reza Tabrizi, Mahmood Moosazadeh, Alireza Razzaghi, Maryam Akbari, Seyed Taghi Heydari, Seyed Habibollah Kavari, Arash Mani, Maryam Kazemi, Kamran Bagheri Lankarani

**Affiliations:** ^*a*^Health Policy Research Center, Institute of Health, Student Research Committee, Shiraz University of Medical Scienc-es, Shiraz, Iran.; ^*b*^Health Sciences Research Center, Faculty of Health, Mazandaran University of Medical Sciences, Sari, Iran.; ^*c*^Safety Promotion and Injury Prevention Research Center, Shahid Beheshti University of Medical Science, Tehran, Iran.; ^*d*^Health Policy Research Center, Institute of Health, Shiraz University of Medical Sciences, Shiraz, Iran.; ^*e*^Faculty Member of Rehabilitation Management Department, School of Rehabilitation, University of Social Welfare and Rehabilitation Sciences,Tehran, Iran.; ^*f*^Research Centre of Psychiatry and Behavioral Sciences, Shiraz University of Medical Sciences, Shiraz, Iran.

**Keywords:** Sleep quality disorder, Iranian driver, Meta-analysis

## Abstract

**Background:**

Sleep Quality Disorder (SQD) plays a major role in road accidents. So, this study was carried out to determine the prevalence of SQD among occupational drivers using systematic review and meta-analysis in Iran.

**Methods:**

All Persian and English articles between January, 2000 and October, 2015 which had reported the SQD prevalence in Iranian drivers by Pittsburgh Sleep Quality Index (PSQI) with cross-sectional design, after the quality evaluation process and achieving the required score, were selected. The heterogenic index of the studies was distinguished by using Cochran (Q) and I2 tests. Based on heterogeneity results, a random effects model was used to estimate pooled prevalence of SQD. Meta-regression was also used to investigate the heterogeneity of suspected factors.

**Results:**

In total, 936 articles were found from national and international databases. Ten articles entered to meta-analysis process, ultimately. Since heterogeneity index suggested that there is a consider-able heterogeneity among the results of primary studies (I-squared = 98.8%, Q= 754.1, p less than 0.001), the overall estimation of SQD among Iranian drivers was conducted using random-effects model and its rate was estimated to be 53.4% (95% CI: 38.9-67.8).

**Conclusions:**

Our study demonstrated that more than half of Iranian drivers have SQD. Identifying the drivers with SQD by periodic examinations and providing advice and health care among occupational drivers could be appropriate solutions for decreasing the accident risks.

## Introduction

Sleep quality disorder (SQD) could have various unde-sirable impacts on mind and body health.^1^ According to the results of current studies, SQD leads to adverse effects such as stress and anxiety, depression, irritability, lack of concentration, fatigue, impatience, loss of performance, cognitive deficit and dysfunction, occupational accidents and errors, and clinical conse-quences such as hypertension, stroke, obesity and increase in risk of diabetes, slowdown in individual’s reactions and decrease in life satisfaction in the individual. ^2-4^

Studying SQD is important due to their high prevalence.^5^ Studies which have been carried out in countries with high income shows that SQD of driver is one of the considerable risk factors in road accidents and increases the road accidents three to six times.^6^ In a study which was conducted in 29,600 drivers in Norway who had an accident, it was reported that SQD among drivers was the effective factor in 3.9% of the driving accidents and this rate increases up to 18.6 percent in night accidents. Also, individuals who had driven for 150 kilometers nonstop were involved in 8.1 percent of accidents leading to injury, and 7.3 percent of them was related to SQD.^7^ However, it is less considered.^5^

There are 700 thousand individuals who have claimed to be occupational drivers in Iran and most of them are forced to work during all hours of the day due to their job conditions and economic situation.^8^ Driving is among careers in which SQD leads to increase in errors and as a result, increase in the risk of accidents.^9^

Considering the high rate of accidents and related risk factors in Iran^10-12^ and the role of SQD in road accidents, conducting a comprehensive research for determining the SQD among occupational drivers in Iran is of importance. Accordingly, this research was designed and implemented in order to study the SQD among Iranian occupational drivers and the factors affecting it, through using the best and most comprehensive method which is systematic review and meta-analysis, so that by determining these impacts on their SQD conditions, the proper context for legal policymaking and health advice for decreasing the driving accidents is provided.

## Methods 

**Search Strategy**

In this study, to find the articles published from January, 2000 to October, 2015 in international and national databases such as SID, Iranmedex, Magiran, Irandoc, PubMed, Google Scholar, Scopus and Web of Science were searched based on the following keywords or their Persian equivalents: ‘Prevalence‘, ‘sleep‘, ‘quality‘, ‘disorder‘, ‘sleep quality‘, ‘sleep disorder‘, ‘sleep quality disorder‘, ‘drivers‘, and ‘Iran‘. Search was carried out by two of the researchers, independently. Also, the references of published articles were examined to increase sensitivity and to select a higher number of researches. The evaluation of the search was conducted randomly by one of the researchers and it was determined that none of the relevant studies was ignored. In addition, the gray literatures were searched in order to access the articles which were not published, and research centers and experts in this field were surveyed for unpublished researches.

**Study Selection **

The full text or the abstract of all articles, records and reports derived from the advanced search were extracted. After excluding the duplicates, the unrelated studies were omitted by screening the title, abstract and full text of the articles, respectively. Subsequently, the related articles were selected. It has to be mentioned that in order to prevent the bias resulting from the republication (transverse and longitudinal publication bias) the researchers also controlled the findings when available to recognize these studies.

**Quality Assessment**

Quality assessment of included articles was performed using STROBE checklist. It consists of questions (12 items) which covers various aspects of methodology such as determining appropriate sample size, study type, sampling method, research populations, data collection method, defining variables and method of studying the samples, data collection tools, statistical tests, research objectives, appropriate reporting of findings and providing the results based on the objectives.^13-15^ Articles with minimum score of 8 to 19 were included in meta-analysis.

**Data Extraction**

The data were extracted based on the title, the first author’s name, year of the study, type of study, sampling method, sample size, language of the article, BMI, average age and SQD prevalence. Then, data were entered into Microsoft Excel spreadsheet. 

**Inclusion Criteria**

All Persian and English articles which had reported the sample size and SQD prevalence by Pittsburgh Sleep Quality Index (PSQI) with cross-sectional design, after the quality evaluation process and achieving the required score, were selected. 

**Exclusion Criteria**

Studies which had not reported the SQD prevalence, along with studies whose sample size was not reported, or the conference and seminars abstracts which did not have the full text, case reports, case-control and interventional studies which do not provide an accurate estimation of the prevalence, and also the studies which could not achieve the minimum evaluation score of quality assessment were excluded. 

**Analysis**

Stata software was used to analyze the data. The prevalence was defined as a proportion: the total patients, divided by the study’s population number. This definition is the same as the binomial distribution definition. The standard assumption in our study was that the prevalence follows a binominal distribution. Therefore, our meta-analysis methods for pooled prevalence based on the inverse variance method, the binomial equation for variance used to estimate the weights of individual studies. Ultimately, the heterogeneity index of the articles was determined by using Cochran (Q) and l2 test. Based on heterogeneity results, the random-effects model was used to estimate the pooled SQD prevalence in Iran. Factors suspected to be heterogenic were assessed using meta-Regression. The sensitivity analysis was also conducted to identify the impact of initial studies on heterogeneity. The point estimates of the prevalence of SQD in Iranian drivers were calculated at confidence interval (CI) of 95% in forest plots. The size of square in this plot shows the weight of each study and also the lines on the both sides indicates the CI of 95%. 

## Results

**Fig. 1:**
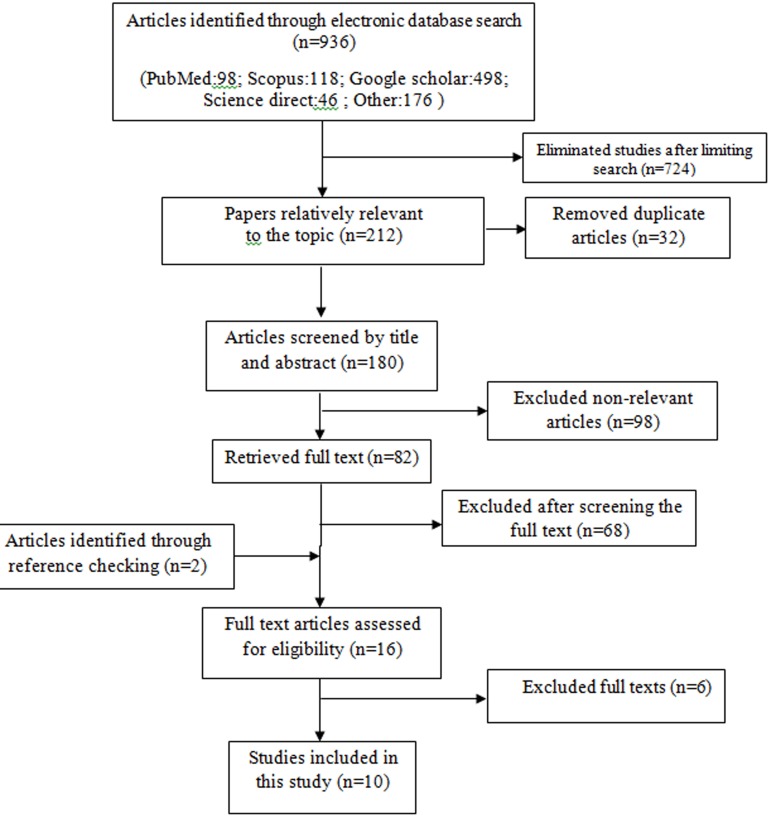
Literature search and review flowchart for selection of primary studies.

Through the initial search, 936 papers were found from national and international databases. After limiting the search strategy and omitting the duplicates due to the overlap of the databases, 180 records were left. Through screening the titles and abstracts, 95 records were determined to be irrelevant. Full text of the 82 remaining articles was studied, and 68 of them were irrelevant. Two articles were added through studying the references. Subsequently, by evaluating the articles quality and inclusion and exclusion criteria, 6 articles were omitted and 10 articles entered the meta-analysis process (Fig. 1).

The research type in 100% of the records entered the study was cross-sectional and the tool (questionnaire) used in 100% of them was PSQI. The sampling method in 8 of them was random and in two cases, the convenient sampling was used. The average age of the studied drivers among the studies entered the meta-analysis varied from 35.4 years in Ebrahimi’s study to 44.1 years in Malek’s study. Also, the BMI was between 25 in Ebrahimi’s study to 26.96 in Mozafari’s study (Table 1).

**Table 1:** Distribution of characteristics of primary studies included in meta-analysis.

Id	First author or corresponding	Publication year	Average age	BMI	Sample size	Prevalence of sleep quality disorder
1	Hassanzadeh^27^	2008	36.7	-	453	73.5
2	Emkani ^28^	2013	40.51	25.4	100	61
3	Effatpanah ^22^	2012	39.2	-	238	85
4	Dehghani ^29^	2015	35.5	25.3	312	27
5	Kakoee ^26^	2010	41.9	25.9	110	78.2
6	Haghighi ^8^	2014	36.66	-	1500	62.3
7	Malek ^20^	2011	44.07	26.4	150	39.3
8	Khanjani ^28^	2012	40.06	25.99	100	61
9	Mozafari ^21^	2014	42.15	26.96	214	19.6
10	Ebrahimi ^19^	2014	35.41	25	312	27.5

**Fig. 2:**
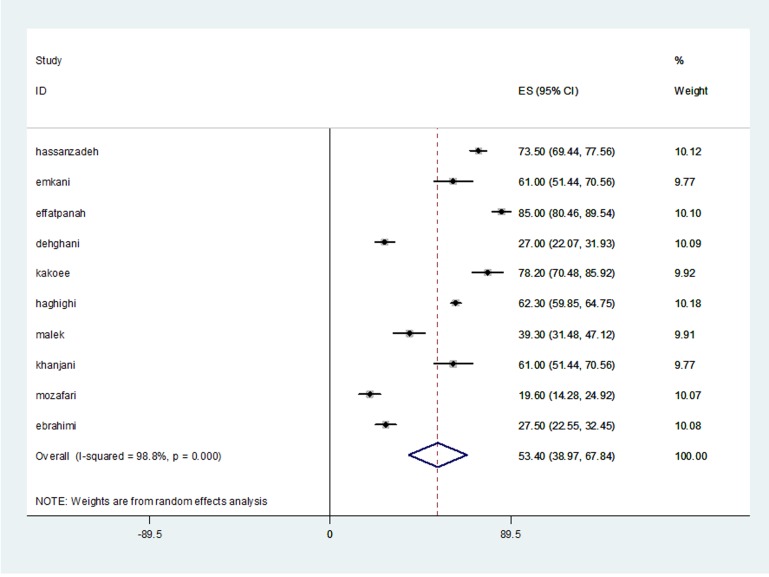
Sleep quality disorder prevalence of the drivers in each study and its overall estimation using random-effects model.

In 10 studies which had the inclusion criteria, the SQD prevalence was studied among 3,489 Iranian drivers. SQD prevalence among the initial studies entered the meta-analysis varied from 19.6% in Mozafari’s study with the sample size of 214 individuals to 85% in ‘Effarpanah’s study with the sample size of 238 individuals. Since there was heterogeneity between the results of initial studies (I-squared = 98.8%, Q = 754.1, p<0.001), the pooled prevalence of SQD was estimated to be 53.4% (95% CI: 38.9-67.8) among Iranian drivers using random-effects model (Table 1& Fig. 2).

**Fig. 3:**
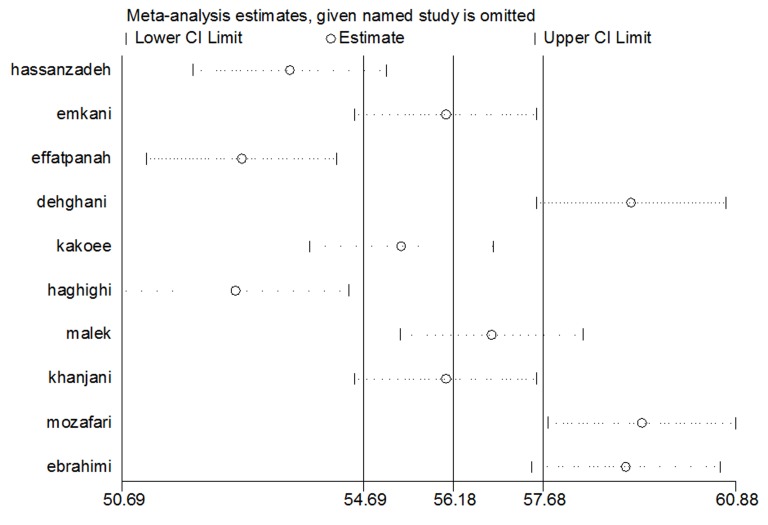
Sensitivity analysis for studying the initial articles impacting the results heterogeneity.

The sensitivity analysis results suggested that 6 studies out of 10 studies had a considerable impact on the heterogeneity among the results (Fig. 3).

Based on meta-Regression results which are shown in Table 2, there is no significant difference between SQD prevalence based on BMI and average age (p>0.05). Accordingly, BMI and average age of the drivers, as the heterogeneity sources, are not among the results of the initial studies entered the meta-analysis. 

**Table 2:** Results of Univariate and Multivariate based on Meta-Regression analysis.

Variable	Univariate		Multivariate	
	Beta Coefficient	P	Beta Coefficient	P
BMI	- 3.5	0.8	-35.5	0.1
Age Average	0.4	0.8	8.2	0.09

## Discussion

Traffic accident in developing countries is the important cause of morbidity and mortality.^16-18^ Considering the significance of sleeping among drivers and its impact on road accidents, higher attention is being paid to the SQD of drivers and its role in accidents.^3^ This research is conducted through meta-analysis method in order to estimate the SQD of Iranian drivers. Ten studies were evaluated in this research. The tool used for studying the SQD prevalence in all the studied researches was PSQI. This questionnaire studies seven components of subjective sleep quality, sleep disorders, efficiency, latency, duration, use of sleep medication and daytime dysfunction. 

In studying the heterogeneity of the primary studies, results suggested that there is a considerable heterogeneity among the results of the primary studies. The sensitivity analysis results suggested that 6 studies out of 10 studies had a considerable impact on the heterogeneity among the results. 

The results from studying the average age of drivers and BMI showed that the average age of the drivers among the studies entered the meta-analysis varied from 35.4 years in Ebrahimi’s study^19^ to 44.1 years in Malek’s study^20^ and the BMI was between 25 in Ebrahimi’s study^19^ to 26.96 in Mozafari’s study. ^21^ Hence, the variables of drivers’ age and BMI were studied as probable factors of forming heterogeneity by meta-Regression analysis. Results from meta-Regression model suggested that these two variables (drivers’ BMI and average age) are not considered as heterogeneity factors. 

Based on the results of meta-analysis, SQD prevalence among Iranian drivers was estimated to be 53.4% with (95% CI: 38.9-67.8). The highest prevalence was related to ‘Efatpanah et al. with 85% (95% CI: 54.46-89.80) which was conducted on 238 intercity bus service drivers, in 2005.^22^ The lowest prevalence was related to the study of Mozafari et al. with 19.60% (95% CI: 92.24-28.14) which was carried out on 214 drivers.^21^ The prevalence of SQD is different in various countries, for instance, according to the U.S. National Highway Traffic Safety Administration, 37% of 1.3 million drivers have had the experience of drowsiness during driving which has been due to the SQD among these drivers. The SQD in the U.K. is reported to be 10-20%.^23^ The difference between observed ratios in various countries could be due to the social, economic and cultural differences. 

SQD in drivers is considered as one of the accident risk factors. The road accident prevalence among drowsy drivers is significantly higher than other drivers,^24^ so that SQDs among drivers lead to 2 to 7 times increase in traffic accidents.^25^ According to U.S. national center on SQDs research, drowsiness leads to death in 36% of accidents and among 42% to 54% of the total accidents is related to drowsiness.^23^ In Malek et al. study in Iran, 25.3% of accidents was attributed to drivers’ drowsiness. ^20^

There are 700 thousand individuals who have claimed to be occupational drivers in Iran and most of whom are forced to work during all hours of the day including drowsiness peak hours, due to their job conditions and economic situation. These working conditions leads to dissatisfaction among drivers.^8^ In some of the conducted studies in Iran, lack of job satisfaction is introduced among the effective factors in drivers’ SQD.^8,26^

Considering the relatively high prevalence of SQD among Iranian drivers, identifying the drivers who are diagnosed with SQD based on periodic examinations for issuing health cards, providing advice and health care could be appropriate solutions for this group of drivers. Screening and detection of drivers diagnosed with SQDs leads to removing related problems and improvement of drivers with SQD and subsequently decrease in the risk of accidents. 

**Acknowledgement: **

We thank Health Policy Research Center of Shiraz University of Medical Sciences for supporting this research financially.
